# Machine-Learning-Based Diabetes Mellitus Risk Prediction Using Multi-Layer Neural Network No-Prop Algorithm

**DOI:** 10.3390/diagnostics13040723

**Published:** 2023-02-14

**Authors:** J. Jeba Sonia, Prassanna Jayachandran, Abdul Quadir Md, Senthilkumar Mohan, Arun Kumar Sivaraman, Kong Fah Tee

**Affiliations:** 1 Department of Data Science and Business Systems, School of Computing, SRM Institute of Science and Technology, College of Engineering and Technology, Kattankulathur, Chennai 603203, India; 2 School of Computer Science and Engineering, Vellore Institute of Technology, Chennai 600127, India; 3 School of Information Technology and Engineering, Vellore Institute of Technology, Vellore 632014, India; 4 Centre for Data Research, Esckimo Robotics Inc., Chennai 600002, India; 5 Faculty of Engineering and Quantity Surveying, INTI International University, Nilai 71800, Malaysia

**Keywords:** diabetes classification, gestational, machine learning, multi-layer neural network

## Abstract

Over the past few decades, the prevalence of chronic illnesses in humans associated with high blood sugar has dramatically increased. Such a disease is referred to medically as diabetes mellitus. Diabetes mellitus can be categorized into three types, namely types 1, 2, and 3. When beta cells do not secrete enough insulin, type 1 diabetes develops. When beta cells create insulin, but the body is unable to use it, type 2 diabetes results. The last category is called gestational diabetes or type 3. This happens during the trimesters of pregnancy in women. Gestational diabetes, however, disappears automatically after childbirth or may continue to develop into type 2 diabetes. To improve their treatment strategies and facilitate healthcare, an automated information system to diagnose diabetes mellitus is required. In this context, this paper presents a novel system of classification of the three types of diabetes mellitus using a multi-layer neural network no-prop algorithm. The algorithm uses two major phases in the information system: the training phase and the testing phase. In each phase, the relevant attributes are identified using the attribute-selection process, and the neural network is trained individually in a multi-layer manner, starting with normal and type 1 diabetes, then normal and type 2 diabetes, and finally healthy and gestational diabetes. Classification is made more effective by the architecture of the multi-layer neural network. To provide experimental analysis and performances of diabetes diagnoses in terms of sensitivity, specificity, and accuracy, a confusion matrix is developed. The maximum specificity and sensitivity values of 0.95 and 0.97 are attained by this suggested multi-layer neural network. With an accuracy score of 97% for the categorization of diabetes mellitus, this proposed model outperforms other models, demonstrating that it is a workable and efficient approach.

## 1. Introduction

Diabetes is a metabolic disease characterized by elevated blood sugar levels and organ damage such as kidney failure [1]. When there is insufficient insulin production or utilization, which causes diabetes, computer programs make it easier to develop an IT system for identifying diseases based on clinical data [2].

Based on genetics and diagnostic criteria [3] as well as the region’s epidemics, the most common type of diabetes in adults is mellitus. Pre-determined factors such as IFG or IGT are used to diagnose diabetes according to the American Diabetes Association (ADA). It can also be diagnosed if the blood glucose level is more than 200 mg/dL based on HbA1c, OGTT, and FPG. Juvenile type 1 diabetes refers to insulin-dependence, and it occurs when the beta cells in the body cannot produce enough insulin. Beta cells vary from infants to adults [4,5,6,7,8]. Other complications such as heart problems, nerve disorders, and kidney-related complications can occur if glucose levels are not properly regulated in the body [9]. Usually, type 1 diabetes occurs under the age of 30, and patients are insulin-dependent, pumping insulin into their bodies.

Type 2 diabetes mellitus is non-insulin-dependent and is otherwise determined as adult diabetes. It occurs due to the loss of secretion of insulin by β cells. Genetics, obesity, and an unhealthy lifestyle will contribute to the increase. Furthermore, type 2 diabetes occurs in middle age. It can also occur prior to gestational diabetes in females and ethnic populations. Additionally, it has an impact on adolescents and children. Based on maternal characteristics during the second and third trimesters of pregnancy as well as biomarkers during the gestation period, gestational diabetes is anticipated. Recent studies have found that pregnant women of diverse ethnicities are at high risk for diabetes [10].

With modern technology, a massive amount of data is recorded, which facilitates the use of machine learning. Doctors are able to analyze a patient’s disease using clinical metrics such as blood pressure and temperature and follow treatments after iterative analysis and refinement [11]. Furthermore, artificial intelligence plays a significant role in fuzzy-based classification and diagnosis of disease using neural networks. Computer-aided comprehension is a factor that makes the situation worse, and the artificial NN ensemble is used for better disease diagnosis [12,13,14]. The authors in [15] used a hybrid neural network algorithm for the IoT-based health care system. The suggested method successfully finds the infiltration in the IoT network. An intelligent system plays a dynamic role in medical analysis based on machine learning techniques, which enhances efficiency and accuracy. The rules are extracted from the knowledge that is fed into an artificial neural network, which can also solve rule-based diagnoses with a fuzzy ANN ensemble.

In this study, we propose a medical information system for diagnosing gestational, type 1, and type 2 diabetes using a multi-layer neural network no-prop algorithm. There are four primary patient categories in the input data (patients), such as normal, type 1 diabetes, type 2 diabetes, and gestational diabetes. With the help of the attribute-selection process, we identify the relevant attributes; then, we train each neural network individually in a multi-layer manner, starting with normal and type 1 diabetics; then, we train normal and type 2 diabetics, and finally, we train healthy and gestational diabetes. An individually trained multi-layer neural network is used to diagnose whether the patient falls into a normal, type 1, type 2, or gestational diabetic category.

The main contributions of the paper are as follows: A diabetes classification system for three kinds of diabetes, such as type 1, type 2, and gestational diabetes is applied. A specialized dataset is created for the experimentation collected over the internet. A multi-layer neural network is used for classification using the no-prop algorithm.

The rest of the paper is organized as follows: the Section 2 examines the related literature, and the Section 3 and Section 4 contain details on the datasets and the proposed multi-layer neural network for classification using the no-prop algorithm. The Section 5 contains the results and discussion of the proposed method, and the Section 6 concludes the proposed method.

## 2. Related Works

This analysis of the literature highlights relevant machine-learning-based research that can quickly identify diabetes. For diabetic patients of type 2, researchers have developed a classification system. Clinical datasets collected from UCHT were used, and knowledge discovery was performed using data mining techniques. When features are selected uniquely, better efficiency is achieved. The condition of type 2 diabetic patients can be predicted using classification techniques such as C4.5. Scientists utilize machine learning algorithms such as support vector machine (SVM), ANN, etc., for the classification of type 2 diabetes. Type 2 diabetes can be better diagnosed and predicted using SVM. The naïve Bayes algorithm offers the highest accuracy among the three classification algorithms used by modern researchers to predict diabetes.

A novel classification method for diabetes diagnosis and classification is proposed in [16]. CGM is used to record and monitor the patient’s glucose level at specific intervals. Clinical datasets were collected from the People’s Hospital of China, and the authors performed analysis on the Chinese ethnic race. Initially, the 17 features were extracted through GSM, and later, variant algorithms of AdaBoost were used as a novel indicator to diagnose and classify diabetes types with experimental results of 90.3% accuracy. Performance indicators such as ACC and MCC were used to evaluate the results.

Authors in [17] determined gestational diabetes, which is a significant parameter in developing type 2 diabetes after delivery. The significant risk factors include an increase in BMI during pregnancy, ethnic race, and age. A comparative analysis was achieved on gestational diabetes mellitus (GDM) and non-GDM. Moreover, a statistical analysis was performed using ANOVA and SPSS. The predictive model was constructed using WEKA software with techniques such as the naïve Bayes classifier and J48. Clinical-related parameters were analyzed using Pearson correlation coefficients. This work shows the metabolomics signature transformation from gestational diabetes to type 2 diabetes. Authors in [18] suggested the SVM machine learning algorithm for the classification of diabetes. SVM uses a large multidimensional space for transforming the input by using the kernel functions. Fourteen diverse attributes were used for the two types of classification schemes based on diagnosed diabetes, undiagnosed diabetes, pre-diabetes, and no diabetes. Performance analysis was carried out by ROC and other cross-validation functions. RBF and linear kernel functions outperformed the best classification schemes.

The authors of the research [19] provided an examination of early diabetes mellitus prediction using machine learning methods. To perform statistical analysis, many health organizations’ data were used. Using the attributes, supervised learning algorithms were employed for classifications and comparative analysis. A new modified approach was applied by selecting optimal features, and a few attributes were eliminated due to smaller correlation values and mapped with the algorithms. Hence, decision tree and random forest algorithms outperformed the others, with a high specificity of more than 98%. In the paper [20], the authors proposed an early diagnosis of diabetes using fuzzy SVM. A dataset with eight attributes was collected from the PID database. Data pre-processing was applied to all eight attributes, and only six attributes were considered for diagnosis. Feature selection and F-score were used to optimize the best features by removing the inappropriate features. Finally, the classification was performed using fuzzy SVM for the prediction of diabetes. A logical regression model of machine learning was proposed for the analysis of HbA1 in diabetes patients with clinical datasets for type 2 diabetes.

## 3. Dataset Preparation

A major part of the research involves building the datasets for the three types of diabetes. To evaluate the proposed classification algorithm, two datasets are constructed based on all three categories of diabetes.

### 3.1. Type 1 Diabetes

Diabetes type 1, or juvenile diabetes, occurs when carbohydrate-based food absorbs energy and retains glucose. Insulin converts glucose into body cells. When the immune system attacks beta cells, type 1 diabetes develops. The main goal of the proposed approach is to extract a dataset that contains diabetic patients’ details; the dataset used for the classification of types of diabetes was obtained from the UCI online library. The data repository has a type 1 diabetes dataset that has eight attributes and class labels ranging from 0 to 1. The data selected are full of females to relate the type 1 dataset with gestational diabetes since gestational diabetes affects only during pregnancy. Type 1 is characterized by symptoms and causes. The dataset contains 768 patient records, with 500 records of positive diabetes class and 268 records of negative diabetes class.

### 3.2. Type 2 Diabetes

The type 2 diabetes datasets contain 19 attributes contributed by the Virginia School of Medicine. Out of 1046 datasets, only 403 were considered based on the attributes. The type 2 dataset was compared with the type 1 dataset, and eight significant parameters were determined for the classification of diabetes.

Considering hypertension increases the risk of type 2 diabetes, parameters such as blood pressure and hypertension are related. Only female subjects were considered for gestational diabetes, which can be considered type 3 diabetes. Gestational diabetes is caused by high blood sugar levels, high blood pressure, and excess amniotic fluid. In the proposed approach, the datasets for gestational diabetes were extracted from type 1 and type 2 datasets after analyzing similar attributes. Based on the characteristics of type 1 and type 2, the attributes of gestational diabetes can be determined.

## 4. Materials and Methods

**Figure 1:**
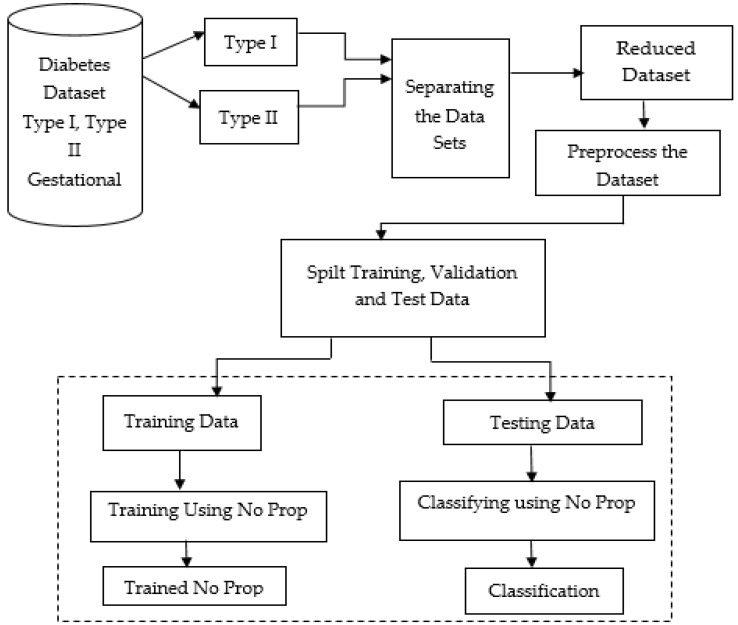
Proposed no-prop multi-layer neural network (NN).

Figure 1 below shows the proposed model, which shows the flow of the no-prop multi-layer neural network.

The datasets of type 1, type 2, and gestational diabetes were fed into the system. Among that, the datasets were separated into type 1 and type 2 datasets. In order to prevent overfitting, the datasets were separated, then reduced, and preprocessed. To make the data better and also to handle missing values, the data were cleaned and normalized after importing. Using a standard ratio for validation, the dataset was split into a training and testing set after preprocessing. With the proposed no-prop algorithm, training and classification were completed separately. A detailed explanation of the proposed method is explained below.

### 4.1. Proposed Techniques for Attribute Selection

The earlier sections of this paper discussed various types of diabetes and the attributes associated with each. Using this data, the proposed approach creates a separate set of datasets, which represents the characteristics of all three types of diabetes. Although each type of diabetes has several attributes, the proposed approach uses only the most interrelated attributes.

A discretization of attributes was applied to categorize them based on the relationships. To discretize the attributes, their count and range were calculated and categorized mainly into four groups. All classes of diabetes attributes were sorted in ascending order based on the minimum and maximum values. The interval was calculated based on the vector and sorted in ascending order for every attribute for diabetes diagnosis of type 1, type 2, and gestational diabetes. Finally, the data were converted to a discretized value that falls under the specified interval. Authors [21] performed a study on the analysis of type 2 diabetes using logistic regression and data mining methods.

As shown in the proposed mathematical model, qualitative analysis and attribute selections were both carried out. The coupled Equations (1) and (2) of the delay system are the formulations mentioned in the equilibrium point as follows also in Equations (1) and (2), we obtain the following:

(1) $$a\left(h\right)=a\left(h-i\right)=a\left(0\right)=a*\forall h$$

(2) $$b\left(h\right)=b\left(h-i\right)=b\left(0\right)=b*\forall h$$

The derivatives are calculated based on time:

(3) $$a\left(h\right)=a\left(h-i\right)and\;a^{\prime }=0$$

Arithmetic operations are applied to both the Equations (4) and (5):

(4) $$b\left(h\right)=b\left(h-i\right)and\;b^{\prime }=0$$

(5) $$0=-x_{1}P^{*}-x_{2}x*b+x_{3}\ldots$$

(6) $$0=y_{1}P^{*}-y_{2}b^{*}$$

(7) $$x_{2}y_{1}P*2+x_{1}y_{2}P^{*}-x_{3}y_{2}=0$$

The equilibrium point (p∗, r∗) will be as follows by solving this quadratic equation:

(8) $$p^{x}=\frac{-x_{1}p_{2}+\sqrt{{\left(x_{1}p_{2}\right)}^{2}+4x_{2}p_{2}x_{3}p_{1}}}{2r_{1}p_{2}}$$

(9) $$r^{*}=\frac{-x_{1}p_{2}+\sqrt{{\left(x_{1}p_{2}\right)}^{2}+4x_{2}p_{2}x_{3}p_{1}}}{2x_{2}p_{2}}$$

$u^{*}\;\geq 0,\;$ and $v^{*}\;\geq 0$ since all parameters are involved and are positive. Thus, the interior equilibrium point $B1=\left(u^{*}\;,\;v^{*}\right)\;$. exists unconditionally. This equilibrium point *B*1 of the model Equations (1) and (2) can be obtained by the linearized model:

Let

b(*t*) = a(*t*) − a∗, s(*t*) = p(*t*) − p∗ (10)

Thus,

(11) $$\frac{du}{dt}=-p_{1}\;u\left(t\right)-p_{2}\;y^{*}\;u\left(t-s\right)-p_{2}x^{*}v\;\left(t-s\right)-p_{2}\;u\left(t-s\right)v\left(t-s\right)-p_{1}x^{*}\;-p_{2}x^{*}y^{*}+p_{3}$$

(12) $$\frac{dv}{dt}=-r_{1}\;u\left(t\right)-r_{2}\;v\left(t\right)+r_{1}x^{*}-r_{2}y^{*}$$

(13) $$\frac{du}{dt}=-p_{1}u\left(t\right)-p_{2}\;y^{*}u\left(t-s\right)-p_{2}\;x^{*}v\left(t-s\right)\;$$

(14) $$\frac{dv}{dt}=-r_{1}u\left(t\right)-r_{2}\;s\left(t\right)$$

Thus, from the obtained linearized model, the characteristic equation is as follows:

(15) $$\Delta \;\left(\;\xi ,s\right)=\;\xi^{2}+p\xi +\left(r\xi +l\right)e^{-\xi s}+m=0\;p=p_{1}+\;r_{1}\;r=p_{2}y^{*}\;l=p_{2}r_{1}x^{*}+p_{2}r_{2}y^{*}\;m=p_{1}r_{2}\;s=0$$

(16) $$\Delta \;\left(\;\xi ,0\right)=\xi^{2}+\left(p+r\right)\xi +l+m=0\;Sum\;of\;roots=\left(p+r\right)\;product\;of\;roots=\left(d+n\right)\;0\;\&\;d+n\;>\;0\;s\;=\;0\;p\;+q\;>\;0,\;\mathrm{and}\;d+n\;>0$$

Hence, for *s* = 0, the equilibrium point *B*1 is locally asymptotically fixed in the lack of time delay if and only if both conditions, i.e., *p* + q > 0 and d + n > 0, hold at the same time. The interior-fixed point *B*1 is locally and asymptotically fixed when *s* = 0 if

(17) $${\left(r_{1}-p_{2}x^{*}\right)}^{2}<4r^{2}\;(p_{1}+p_{1}\;y^{*}\;)$$

*s* ≠ 0, if *ξ* = *i*ω then ω (ω > 0)

Now, for *s* ≠ 0, if *ξ* = *i*ω is a root of a specific Equation (9a), then ω (ω > 0) will fill the equations given as

ω*r* sin ω*s* + *l* cos ω*s* = ω^2^ − *m*

ω*r* cos ω*s* − *l* sin ω*s* = −*p*ω

Squaring both the above equations, we obtain the following:

ω^2^*r*^2^ sin^2^ ω*s* + *l*^2^ cos^2^ ω*s* + 2ω*rl* sin ω*s* cos ω*s* + ω^2^*r*^2^ cos^2^ ω*s* + *l*^2^ sin^2^ ω*s*

−2ω*rl* sinω*s* cos ω*s* = ω^4^ + *m*^2^ − 2ω2*m* + *p*^2^ω^2^

⟹ ω^2^*r*^2^(sin^2^ ω*s* + cos^2^ ω*s*) + *l*^2^(sin^2^ ω*s* + cos^2^ ω*s*) = ω^4^ + *m*^2^ − 2ω2*m* + *p*^2^ω^2^

⟹ −ω^2^*r*^2^ − *l*^2^ + ω^4^ + *m*^2^ − 2ω2*m* + *p*^2^ω^2^ = 0

⟹ ω^4^ + (*p*^2^ − *r*^2^ − 2*m*)^2^ + *m*^2^ − *l*^2^ = 0

ω^2^ = [(−(*p*^2^ − *r*^2^ − 2*m*)) ± √(*p*^2^ − *r*^2^ − 2*m*)^2^ − 4(*m*^2^ − *l*^2^)]/2

$$p^{2}-r^{2}-2m>0\;\&\;m^{2}-l^{2}>0z_{2}^{0}\;\mathrm{if}\;l^{2}-m^{2}<0$$

If *m*^2^ − *l*^2^ > 0, *r*^2^ − *p*^2^ − 2*m* > 0, and (*r*^2^ − *p*^2^ − 2*m*)^2^ > 4(*m*^2^ − *l*^2^)

Then, there occurs two positive solutions ω± ^2^.

The needed and necessary requirements for lack of uncertainty are caused due to time delay.

### 4.2. Class Association Rule

Attribute weightage: The proposed approach of categorizing diabetes accounts for the many forms of diabetes. The attributes that describe the traits of each type of diabetes are the primary characteristics connected with the classification procedure. The proposed method puts forward a technique for selecting the attributes based on their weightage. An attribute weight calculation method was adopted based on the association of each attribute with the classes derived from the dataset. The attribute weightage calculation process mainly considers two data matrices, which are formed by the attributes of patient ID and class values. The attributes and the patient list form the initial matrix, and the second matrix is created by the class labels and the attribute lists.

The attribute weight is calculated by finding the frequency of each attribute in both matrices. The relevance of each attribute regarding the matrices is calculated independently and then dependently. The above process determines the importance of an attribute regarding its class. The weight of the attribute is derived mainly based on the classes, i.e., how an attribute is associated with a particular category, which will help the proposed method to classify the four different categories of diabetes. The initial process of the attribute weight calculation phase is the comparison between the two matrices. Initially, the attribute frequencies regarding the classes are calculated for finding the importance of the attributes. Once the class-based frequency is calculated, then the attribute weight is analyzed based on the first matrix. The weight of the attribute is associated with its presence and absence in both matrices:

(18) c1:a1⇒ a1 in c1

The above expression shows the relevance of attribute a1 in class1 based on the presence of attribute a1 in the data matrix one. Similarly, the presence of attributes is calculated based on their appearance in matrix one. The c1 : a1 value gives an integer value, which is obtained as the count of a1 present in matrix one in the relevant class c1:

(19) $$c1\;:\;a1\;\Rightarrow \;a1\;\mathrm{in}\;c1=\;\mathrm{Countc}1\left(a1\right)$$

The presence and absence of the attribute are calculated consecutively for the calculation of the attribute length. Thus, from the above-calculated values, the weight of attribute a1 is calculated as per Equation (18):

(20) $$\mathrm{weight}\left(a\;1\right)=\;\mathrm{Countc}1\left(a1\right)\mathrm{Countc}1\left(a1\;\omega \right)$$

The weightage value provides the relevance of attribute a1 based on class1. Similarly, the weight of each attribute based on each class was calculated, and the attributes having the highest weightage were chosen for improving the efficiency of the proposed classification method.

**No-Prop Algorithm:** Diabetes dataset_1 and dataset_2 use the same procedure to calculate attribute strength. *Generating Algorithm Begin*

**Step 1**: Import the dataset_1.

**Step 2.** The dataset_1 is normalized (i.e., converted to (0–1) range) by dividing each element with the maximum value of that column.

**Step 3.** The dataset_1 is discretized by assigning value 1 to those elements for which the value is between 0 and 0.25, value 2 to those elements for which the value is 0.25 to 0.5, value 3 to those elements for which the value is between 0.5 and 0.75, and value 4 to those elements for which the value is between 0.75 and 1.

**Step 4.** To calculate attribute strength.

(i) We form a matrix of the following:
  Dimension number of rows = number of discrete levels X number of types of diabetes number of columns = number of attributes;
(ii) (a) Numerator = determine the number of times the value 1 occurred in the first column and 1 occurred in the same row of the last column (i.e., type column);
  (b) Denominator = determine the number of times the value 1 occurred in the first column and 1 did not occur in the same row of the last column;
  matrix (1,1) = numerator/denominator;
  (c) Numerator = determine the number of times the value 1 occurred in the first column and 2 occurred in the same row of the last column;
  (d) Denominator = determine the number of times the value 1 occurred in the first column and 2 did not occur in the same row of the last column;
  matrix (2,1) = numerator/denominator;
  (e) Numerator = determine the number of times the value 1 occurred in the first column and 3 occurred in the same row of the last column (i.e., type column);
  (f) Denominator = determine the number of times the value 1 occurred in the first column and 3 did not occur in the same row of the last column;
  matrix (3,1) = numerator/denominator;
  (g) Numerator = determine the number of times the value 1 occurred in the first column and 4 occurred in the same row of the last column;
  (h) Denominator = determine the number of times the value 1 occurred in the first column and 4 did not occur in the same row of the last column;
  matrix (4,1) = numerator/denominator;
  (i) Numerator = determine the number of times the value 2 occurred in the first column and 1 occurred in the same row of the last column;
  (j) Denominator = determine the number of times the value 2 occurred in the first column and 1 did not occur in the same row of last column;
  matrix (5,1) = numerator/denominator;
  (k) Numerator = determine the number of times the value 2 occurred in the first column and 2 occurred in the same row of the last column;
  (l) Denominator = determine the number of times the value 2 occurred in the first column and 2 did not occur in the same row of the last column;
  (m) Repeat the procedure in step (ii), taking the second column instead of the first column to fill the second column of the matrix.

**Step 5.** Find out the maximum value in each column of the resulting matrix. This is the attribute strength of the attribute that corresponds to that column.

**Step 6.** Discard two columns for which the attribute strength is the lowest.

**Step 7.** Take 80% of the rows at random from the resulting dataset for training.

**Step 8.** Take the remaining 20% of the rows of the dataset for testing.

*End*

### 4.3. Classification of Diabetes Types Using Multi-Layer Neural Network

The approach concentrates on classifying the different types of diabetes by associating different attributes possessed by the different datasets. As discussed in the above section, the proposed method creates four sets of data by associating the attributes. The main objective of those datasets is the reduction of an attribute. The most appropriate attribute among them is selected according to their priority and frequency. Once the characteristics are optimized, then the process of classifying the different types has to be subjected. The proposed approaches consider the neural network method for classification. The classification phase includes mainly two processes: the training phase and the testing phase.

Figure 1 illustrates the proposed architecture of the no-prop multi-layer neural network to classify the different types of diabetes. After the attribute weights are calculated, the attributes are used for training the three neural networks. Later in the training phase, the data to be tested are subjected initially to the normal/type1 NN. If normal data result, then it is subject to the normal/type 2 NN; if again normal, the data are subject to the normal/gestational diabetes. The multi-layer architecture of the neural network will provide improved efficiency for the classification process.

### 4.4. Training Phase

The input layer of the training phase contains eight attributes from the PIMA Indian diabetes dataset, the output layer contains two values for each of the three neural networks, and the class values of each of the four data types will be 1 for normal individuals without diabetes, 2 for type 1 diabetes patients, 3 for type 2 diabetes, and 4 for gestational diabetes.

**Figure 2:**
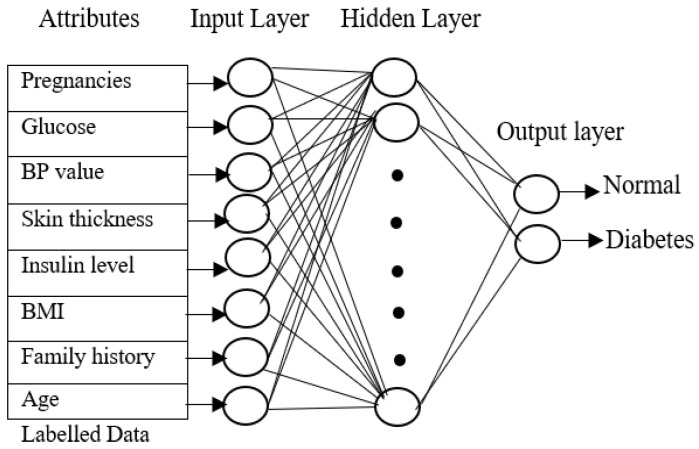
Neural network training phase.

Figure 2 shows the neural network with eight attributes of type 1 diabetes, and the same network can be used by the proposed approach for classifying diabetes for type 2 diabetes with pre-processed attributes. The key objective of the training phase is to compute generalized values for the hidden layers. All the layers are connected to each other to form a network. The neuron is activated by a transmitted signal and has an activation function that acts as a threshold. The weights are found through the activation function. The hidden layer becomes stable only when the neural network derives the least error value from the training dataset. A fast-supervised learning algorithm, scaled conjugate gradient (SCG), reduces the error value in the network. The SCG will continue up to the level in which the hidden layers obtain stable value. Similarly, all three neural networks are trained with the related diabetes dataset, and all the hidden layer values are calculated.

### 4.5. Testing Phase

**Figure 3:**
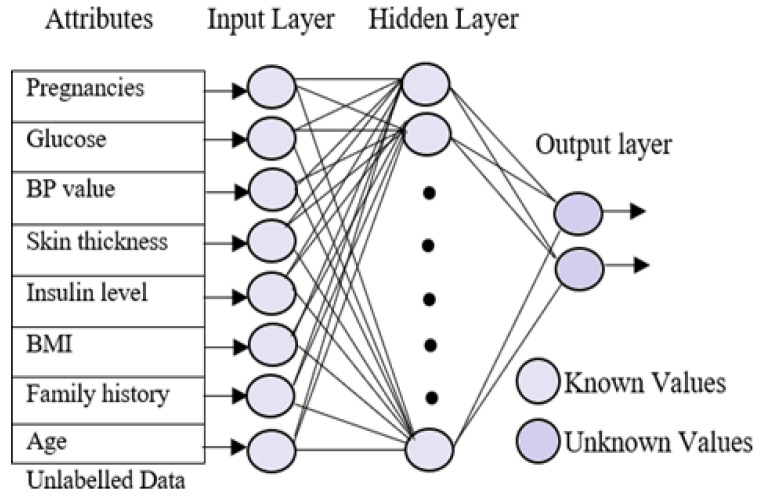
Neural network testing phase.

A sequential testing procedure for classifying the data based on the different diabetes types was applied. In the testing phase, the multi-layer neural network was trained using the patient’s known data. The trained neural network uses three different layers, which are similar to the neural training network but with minor changes. The input layer in the testing phase is identical to the training phase. The hidden layers go through significant changes because they obtains the known values from the training phase. On the other hand, the output layer is empty here in the testing phase. Figure 3 represents the processing of the testing phase. The aim of the testing phase is to classify the unlabeled data into different groups based on the diabetes types. The testing phases use three neural networks, which are trained for different types of diabetes, namely normal, type 1, type 2, and gestational diabetes. Initially, the unlabeled data were tested for type 1 diabetes using the first neural network. The resultant data that were not listed under diabetes, i.e., the normal data, were selected and subjected to the second neural network for finding the possibility of diabetes type 2 in the data. Gestational diabetes was tested separately during the gestation period because it affects only pregnant ladies.

## 5. Experimental Results

Experimental diagnosis of the research study for the diabetes classification method is discussed. It is based on two types of diabetic datasets that were collected from the web. The experiments are implemented in MATLAB, and the toolkit used is the neural network toolbox. To reduce the error value, scaled conjugate gradient was used. We used MATLAB’s training with mean square as the performance function. The Sugeno fuzzy inference system uses the fuzzy logic toolbox for classification.

### 5.1. Dataset Description

The proposed approach uses two different datasets for the classification process according to the various categories of diabetes types, such as type 1, type 2, and gestational diabetes. The datasets used for the type 1 diabetes classification were obtained from the UCI machine learning repository. The Pima Indian Diabetes (PID) dataset is formerly from the National Institute of Diabetes and Digestive and Kidney Diseases; it contains the data of 768 diabetes datasets with 500 positive subjects and 268 negative subjects. Dataset 1 includes three different diabetes types with all common attributes, whereas dataset 2 includes three diabetes types with unique attributes for classification. The type 2 diabetes dataset of 1046 individual data with 19 original attributes contributed by the Virginia School of Medicine was considered. As gestational diabetes is also included in the classification, only 403 datasets were taken, with few attributes found for both type 2 and gestational diabetes by comparing it with the eight attributes of type 1 diabetes.

### 5.2. Neural Network Parameters, Fuzzy Parameters, and Training Algorithm

The neurons are activated through a transmitted signal and also have an activation function that acts as a threshold. The training algorithm used for multi-layer perception neural network (MLPNN) is the scaled conjugate gradient algorithm (SCG). The learning rate was set so that the slopes did not diverge. The neural networks were iterated for epochs with learning rates. The parameters related to the neural network and fuzzy are given in Table 1 and Table 2, respectively. Additionally, Table 2 gives the fuzzy parameters taken for the fuzzy classifier. Here, we used the genfis classifier for fuzzy classification, as there are more than six inputs. From Table 3, we can identify those five hidden layers that were utilized along with the input and output layers.

### 5.3. Performance Analysis

This section discusses the performance analysis of the proposed classification of diabetes types. Specificity, sensitivity, and accuracy are discussed. The evaluation parameters were calculated by testing them with the collected datasets. The performance evaluation was conducted on two datasets that were processed according to the proposed approach. The datasets were tested using the methods of the proposed approach for analyzing the specificity, sensitivity, and accuracy of the proposed approach. The performance evaluation conducted in this section also uses a comparison with an existing work, which is a classification process based on the fuzzy method. The classification is based on three classes: class 2 represents type 1 diabetes; class 3 represents type 2 diabetes; and class 4 represents gestational diabetes. The analysis based on the proposed classification method is shown in the following graphs.

The network’s performance was measured by mean square error (MSE) obtained in epoch 196 with the best performance validation of 0.001428 for gestational diabetes. The error values were taken, they were squared, and their mean value was calculated. The fuzzy inference system maps the given input to output using fuzzy rules and operators. In our proposed system, the logical operator AND and OR method were used. The main workings of the system are fuzzification, inference, and defuzzification. There are two components of FIS: the Mamdani and Sugeno type. We chose the Sugeno model for our work, as the output depends on the input variable. As we have eight multiple attributes as input, the Gaussian membership function was used with crisp value for all the variables individually, and they were fed into the FIS editor. The genfis model was used when there were more than six inputs. To define the membership function genfis uses subtractive clustering and grid partitioning. The genfis returns a single-output Sugeno fuzzy inference system.

The diabetes diagnosis and classification were performed either for type 1, type 2, or both type 1 and type 2. Our neural network architecture focuses on the classification of type 1, type 2, and gestational diabetes. Hence, a multi-layer neural network architecture is proposed.

### 5.4. Evaluation Metrics

**Figure 4:**
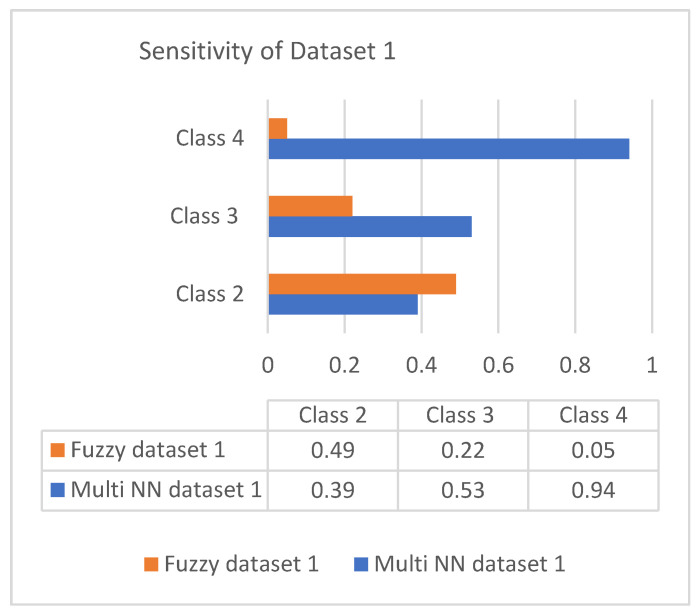
Sensitivity of dataset 1.

**Figure 5:**
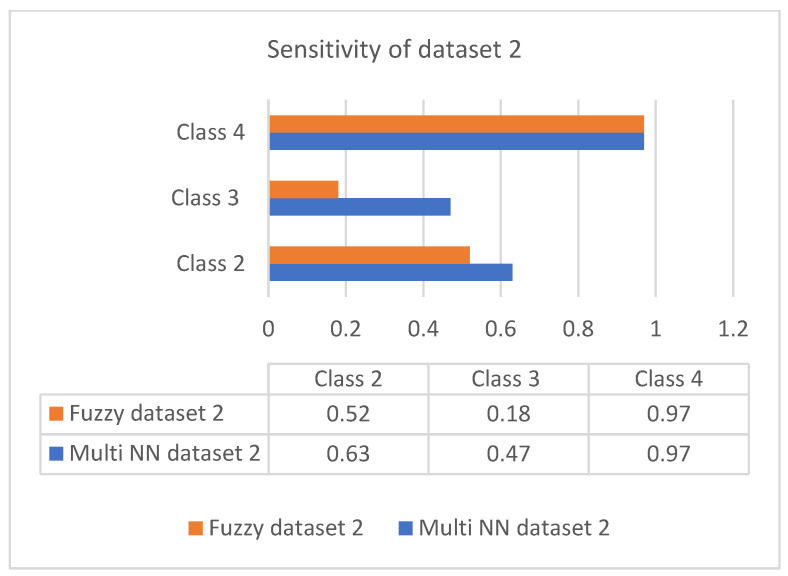
Sensitivity of dataset 2.

Sensitivity is the process of classifying positive cases correctly, and specificity is the process of classifying negative instances correctly. The confusion matrix for classification is composed of true positive (TP), true negative (TN), false positive (FP), and false negative (FN). The accuracy is measured with measures of sensitivity and specificity. Measures were performed on both the datasets and graphically represented in bar charts. Figure 4 and Figure 5 represent the analysis of the proposed approach based on parameter sensitivity. The study from the graphs shows that in both cases, the sensitivity value varies in nature. In the case of dataset 1, the sensitivity variation shows an incremental nature, while in dataset 2, it shows a zigzag view. The results of sensitivity show that most of the patients are affected by type 2 diabetes. Since class 3 represents gestational diabetes, it is not taken into account. The comparison of the sensitivity values with the multi-layer neural network and fuzzy method shows considerable differences. In both datasets, the proposed method shows more sensitivity than the fuzzy approach. In this case, in dataset 1, the fuzzy method shows slightly better performance for class 2. Furthermore, it differs in specificity from the existing method, and a significant difference is shown only in class 2 and class 3 of dataset 1. In both datasets, the response of specificity shows an incremental nature.

**Figure 6:**
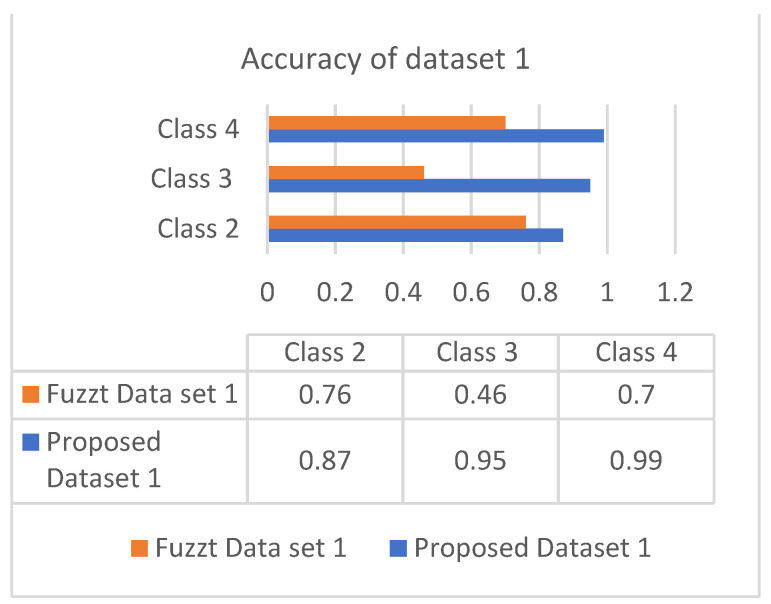
Accuracy of dataset 1.

**Figure 7:**
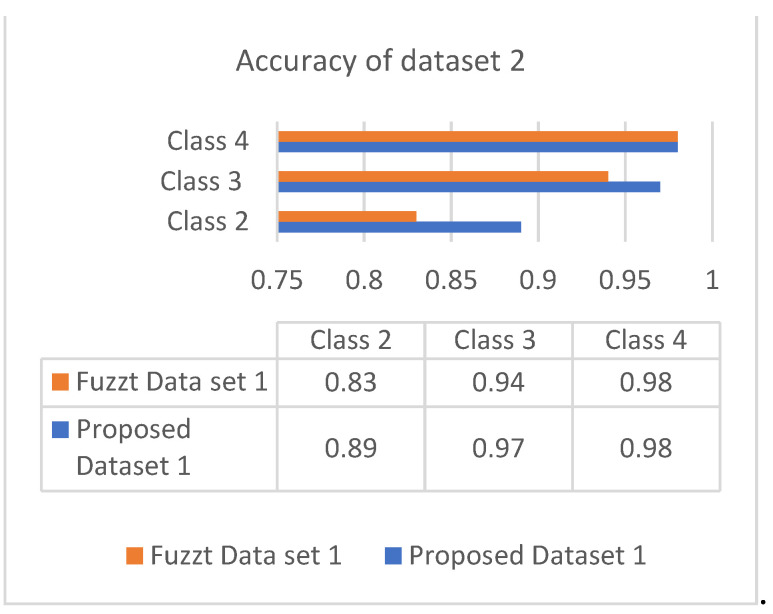
Accuracy of dataset 2.

The accuracy is the combinational result of the parameters’ specificity and sensitivity according to their responses in the datasets. Figure 6 and Figure 7 show the responses of efficiency based on the datasets.

Both datasets produce an incremental way of response based on the classes. The analysis showed that most of the patients are correlated with diabetes of type 2, which occurs in adult age. The values of different analyses are shown in Table 4 and Table 5.

**Figure 8:**
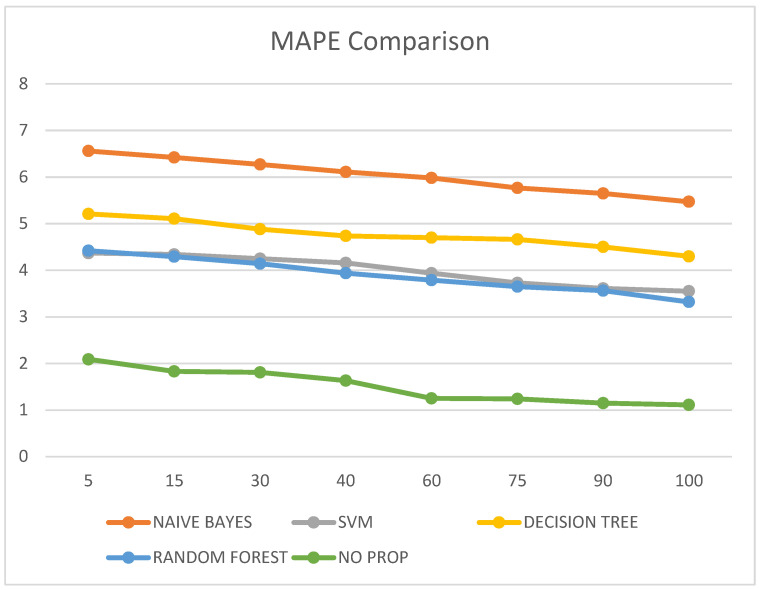
MAPE comparison.

It was also observed that the MAPE value of the proposed no-prop algorithm is lower than any of the other algorithms such as naïve Bayes, SVM, decision tree, and random forest, with a mean error percentage of only 1.11% on the testing data. The MAPE values are illustrated in Table 6 and visualized in Figure 8.

## 6. Discussion and Future Work

In this paper, we present a novel no-prop algorithm for the classification of the three types of diabetes mellitus using a multi-layer neural network. A multi-layer neural network was trained in each phase using the attribute-selection process to identify the relevant attributes for each patient. Each layer of the neural network was trained separately, beginning with normal and type 1 diabetes, then normal and type 2 diabetes, and finally healthy and gestational diabetes. Using a multi-layer neural network, classification is made more efficient. A confusion matrix was developed to generate experimental analysis and performance data concerning diabetes classifications. This proposed multi-layer neural network achieved the highest specificity and sensitivity values of 0.95 and 0.97, respectively. Based on the accuracy score, this model had an edge over other models, with a score of 97% for diabetes mellitus classification. Future work can be carried out by integrating semantic methods and disease diagnosis after classification. The data can be collected from local health authorities, hospitals, and laboratories.

Using a local dataset for future research can have major implications, according to a preliminary investigation. By integrating sensor technologies such as body area networks, data from local sources will be collected in the future. By means of customized software, health data will be collected and analyzed thoroughly. The normalized data will serve as a basis for on-site expert guidance and accurate predictions by a medical professional.

## Figures and Tables

**Table 1:** Multi-layer neural network parameters.

Neural Network	Epoch	No. of Iterations	Error	Training Algorithm	Number of Hidden Layers	Nodes
ML-NN1 (normal/type1 NN)	86	106	0.089677	Scaled conjugate gradient algorithm	5	(11:50:25:5:1)
ML-NN2 (normal/type2 NN)	88	106	0.009476	Scaled conjugate gradient algorithm	5	(11:50:25:5:1)
ML-NN3 (normal/gestational NN)	196	216	0.001428	Scaled conjugate gradient algorithm	5	(11:50:25:5:1)

**Table 2:** Fuzzy parameters.

FIS Type	Sugeno
AND method and Implication	prod
OR method and Aggregation	probor
Membership function	Gaussian
Defuzzification	Wtaver

**Table 3:** Comparison of diagnosis and classification results by different authors.

Authors	Diabetes Type	Classifier	Accuracy %
Hang	Type 2	Naïve Bayes SVM	96 75.30
Deepti	Type 2	Naïve Bayes Decision tree	67.10 74.82
Sneha	Type 1 and Type 2	Naïve Bayes	83
Yu	Type 2	SVM	84.5
Lukmantoa	DM	Fuzzy SVM	90
Allalou	GDM	Decision tree	84

**Table 4:** Classification of diabetes for dataset 1.

Parameters	Dataset 1		
	Class 2	Class 3	Class 4
Sensitivity	0.39	0.53	0.94
Specificity	0.95	0.95	0.96
Accuracy	0.87	0.95	0.99

**Table 5:** Classification of diabetes for dataset 2.

Parameters	Dataset 2		
	Class 2	Class 3	Class 4
Sensitivity	0.63	0.47	0.97
Specificity	0.93	0.99	0.96
Accuracy	0.89	0.97	0.98

**Table 6:** MAPE comparison of different classification techniques at multiple epochs.

Epoch Count	Naïve Bayes	SVM	Decision Tree	Random Forest	No-Prop
5	6.56	4.37	5.21	4.42	2.09
15	6.42	4.34	5.11	4.29	1.83
30	6.27	4.25	4.88	4.14	1.81
40	6.11	4.16	4.74	3.94	1.63
60	5.98	3.94	4.70	3.79	1.25
75	5.77	3.73	4.66	3.65	1.24
90	5.65	3.61	4.50	3.56	1.15
100	5.47	3.55	4.30	3.32	1.11

## Data Availability

Not applicable.

## References

[1] Abhari S., Kalhori S.R.N., Ebrahimi M., Hasannejadasl H., Garavand A. (2019). Artificial intelligence applications in type-2 diabetes mellitus care: Focus on machine learning methods. Healthc. Inform. Res..

[2] Ning F.F., Osborne X., Cox L.R., Gunderson B.J., Wheeler M.B. (2016). A predictive metabolic signature for the transition from gestational diabetes mellitus to type-2 diabetes. Diabetes.

[3] Mujumdar A., Vaidehi V. (2019). Diabetes prediction using machine learning algorithms. Procedia Comput. Sci..

[4] Andrews R., Diederich J., Tickle A.B. (1995). Survey and critique of techniques for extracting rules from trained artificial neural networks. Knowl. Based Syst..

[5] Hasan M.K., Alam M.A., Das D., Hossain E., Hasan M. (2020). Diabetes prediction using ensembling of different machine learning classifiers. IEEE Access.

[6] Joshi T.N., Chawan P.P.M. (2018). Diabetes prediction using machine learning techniques. Int. J. Eng. Res. Appl..

[7] Chiu S. (1994). Fuzzy model identification based on cluster estimation. J. Intell. Fuzzy Syst..

[8] Yahyaoui A., Jamil A., Rasheed J., Yesiltepe M. A decision support system for diabetes prediction using machine learning and deep learning techniques. Proceedings of the 2019 1st International Informatics and Software Engineering Conference (UBMYK).

[9] Cunningham P., Carney J., Jacob S. (2000). Stability problems with artificial neural networks and the ensemble solution. Artif. Intell. Med..

[10] Dutta D., Paul D., Ghosh P. Analysing feature importances for diabetes prediction using machine learning. Proceedings of the 2018 IEEE 9th Annual Information Technology, Electronics and Mobile Communication Conference (IEMCON).

[11] Magoulas G.D., Prentza A. (2001). Machine learning in medical applications. Mach. Learn. Its Appl..

[12] Huang Y., McCullagh P., Black N., Harper R. (2007). Feature selection, and classification model construction on type-2 diabetic patients’ data. Artif. Intell. Med..

[13] Sarwar M.A., Kamal N., Hamid W., Shah M.A. Prediction of diabetes using machine learning algorithms in healthcare. Proceedings of the 2018 24th International Conference on Automation and Computing (ICAC).

[14] Zou Q., Qu K., Luo Y., Yin D., Ju Y., Tang H. (2018). Predicting diabetes mellitus with machine learning techniques. Front. Genet..

[15] Ali A., Almaiah M.A., Hajjej F., Pasha M.F., Fang O.H., Khan R., Teo J., Zakarya M. (2022). An Industrial IoT-Based Blockchain-Enabled Secure Searchable Encryption Approach for Healthcare Systems Using Neural Network. Sensors.

[16] Kavakiotis I., Tsave O., Salifoglou A., Maglaveras N., Vlahavas I., Chouvarda I. (2017). Machine learning and data mining methods in diabetes research. Comput. Struct. Biotechnol. J..

[17] Kharroubi A.T., Darwish H.M. (2015). Diabetes mellitus: The epidemic of the century. World J. Diabetes.

[18] Arunachalam P., Janakiraman N., Rashid J., Kim J., Samanta S., Naseem U., Sivaraman A.K., Balasundaram A. (2022). Effective classification of synovial sarcoma cancer using structure features and support vectors. Comput. Mater. Contin. (CMC).

[19] Kononenko I. (2001). Machine learning for medical diagnosis: History, state of the art and perspective. Artif. Intell. Med..

[20] Saru S., Subashree S. (2019). Analysis and prediction of diabetes using machine learning. Int. J. Emerg. Technol. Innov. Eng..

[21] Khanam J.J., Foo S.Y. (2021). A comparison of machine learning algorithms for diabetes prediction. ICT Express.

